# Fine-Tuning the TGFβ Signaling Pathway by SARA During Neuronal Development

**DOI:** 10.3389/fcell.2020.550267

**Published:** 2020-09-03

**Authors:** Victoria Rozés-Salvador, Carlos Wilson, Cristina Olmos, Christian Gonzalez-Billault, Cecilia Conde

**Affiliations:** ^1^Instituto de Investigación Médica Mercedes y Martín Ferreyra INIMEC-CONICET-UNC, Córdoba, Argentina; ^2^Instituto de Ciencias Básicas, Universidad Nacional de Villa María (UNVM), Córdoba, Argentina; ^3^Instituto Universitario de Ciencias Biomédicas de Córdoba (IUCBC), Córdoba, Argentina; ^4^Department of Biology, Faculty of Sciences and Department of Neurosciences, Faculty of Medicine, Universidad de Chile, Santiago, Chile; ^5^Geroscience Center for Brain Health and Metabolism, Santiago, Chile; ^6^The Buck Institute for Research on Aging, Novato, CA, United States

**Keywords:** TGFβ, Smad Anchor for Receptor Activation (SARA), neurons, development, axon, migration, endosomes

## Abstract

Neural development is a complex process that involves critical events, including cytoskeleton dynamics and selective trafficking of proteins to defined cellular destinations. In this regard, Smad Anchor for Receptor Activation (SARA) is an early endosome resident protein, where perform trafficking- associated functions. In addition, SARA is also involved in cell signaling, including the TGFβ-dependent pathway. Accordingly, SARA, and TGFβ signaling are required for proper axonal specification and migration of cortical neurons, unveiling a critical role for neuronal development. However, the cooperative action between the TGFβ pathway and SARA to this process has remained understudied. In this work, we show novel evidence suggesting a cross-talk between SARA and TGFβ pathway needed for proper polarization, axonal specification, growth and cortical migration of central neurons both *in vitro* and *in vivo*. Using microscopy tools and cultured hippocampal neurons, we show a local interaction between SARA and TβRI (TGFβ I receptor) at endosomes. In addition, SARA loss of function, induced by the expression of the dominant-negative SARA-F728A, over-activates the TGFβ pathway, most likely by preserving phosphorylated TβRI. Consequently, SARA-mediated activation of TGFβ pathway impacts on neuronal development, promoting axonal growth and cortical migration of neurons during brain development. Moreover, our data suggests that SARA basally prevents the activation of TβRI through the recruitment of the inhibitory complex PP1c/GADD34 in polarizing neurons. Together, these results propose that SARA is a negative regulator of the TGFβ pathway, being critical for a proper orchestration for neuronal development.

## Introduction

The neuronal development is a complex process that begins early at the embryonic life. After differentiation, post-mitotic neurons undergo progressive morphological transformations to specify their somato-dendritic and axonal compartments; a process called “the establishment of polarity” (Dotti et al., 1988; Cid-Arregui et al., 1995; Caceres et al., 2012). Therefore, the coordination of several events are needed, including the selective transport of polarity determinants by the endosomal trafficking machinery (Sann et al., 2009; Falk et al., 2014; Takano et al., 2014; Mestres et al., 2016), local segregation of proteins in axon and dendrites (Chuang et al., 2005; Arimura and Kaibuchi, 2007; Funahashi et al., 2020) and a highly dynamic cytoskeleton of microtubules and actin microfilaments (Conde and Caceres, 2009; Wilson et al., 2015). Accordingly, several small GTPase proteins belonging to the Rab (Ras-associated binding) family, controlling intracellular transport, contribute to neuronal polarization (Villarroel-Campos et al., 2016); nevertheless, additional partners are needed. In this regard, Smad Anchor for Receptor Activation (SARA), an early endosome (EE) binding protein, regulates endosomal trafficking and axonal growth of neurons (Tsukazaki et al., 1998; Arias et al., 2015; Mestres et al., 2016). Although the suppression of SARA impairs endosomal dynamics and neuronal development (Arias et al., 2015; Mestres et al., 2016), the mechanistic aspects underlying this effect are still missing.

At endosomes, SARA performs several trafficking functions, including sorting and recycling of membrane proteins such as the Transferrin receptor (TfR) and Rhodopsin (Hu et al., 2002; Chuang et al., 2007; Arias et al., 2015), endosomal segregation during cell division (Coumailleau et al., 2009; Kressmann et al., 2015) and distribution to intracellular compartments (Arias et al., 2015).

However, non-trafficking roles of SARA have also emerged. In fact, SARA participates in cell signaling regulation, including the Notch/Delta pathway in sensory organs of *D. melanogaster* (Coumailleau et al., 2009; Loubery et al., 2014) and both the Epidermal Growth Factor (EGF) and the Transforming Growth Factor (TGFβ)-dependent pathway in epithelial cells, among others (Tsukazaki et al., 1998; Wu et al., 2000; Kostaras et al., 2013, 2014; Rozes-Salvador et al., 2018). Accordingly, TGFβ signaling demands a precise coordination between ligands, receptors, regulatory proteins and transcriptional factors to control proliferation, differentiation, metabolism and apoptosis (Wrana et al., 1994; Shi and Massague, 2003; Wharton and Derynck, 2009; Akhurst and Hata, 2012; David and Massague, 2018). For this aim, SARA recruits Smad2 and Smad3 to the TGFβ type I receptor (TβRI), enabling their phosphorylation and nuclear translocation for transcriptional control (Wrana et al., 1994; Tsukazaki et al., 1998; Panopoulou et al., 2002; Tang et al., 2015). However, SARA is also able to down-regulate this pathway. In both primary cultures of epithelial cells and cell lines, SARA binds to PP1c (the catalytic unit of protein phosphatase 1), which in turns assembles with Smad7 and GADD34; a complex able to dephosphorylate - and inactivate –TβRI (Attisano and Wrana, 2002; Shi et al., 2004; Tang et al., 2010). Therefore, a dual role for SARA has been described, although its precise regulation and impact on neuronal development remain unexplored.

TGFβ plays central roles for the physiology of the nervous system, including inflammatory response modulation, neural commitment, development and progression of several neurodegenerative diseases (van der Wal et al., 1993; Mogi et al., 1995; Vogel et al., 2010; Yi et al., 2010; Meyers and Kessler, 2017). Accordingly, TGFβ also promotes neuronal polarity acquisition and corticogenesis, being critical for brain and neuronal development (Yi et al., 2010). Nevertheless, the regulation of TGFβ signaling by SARA during this process has been understudied.

Based on *in vitro* and *in vivo* assays, we show in this work that SARA allows polarization, axonal growth, and migration of cortical neurons by modulating the TGFβ signaling, a phenomenon mediated by the recruitment of the inactivating complex PP1c/GADD34. Our data suggests that SARA is an endogenous regulator able to fine-tune the TGFβ pathway for a proper neuronal development in time and space.

## Results

### Expression and Interaction of SARA and TβRI in Polarizing Neurons

**FIGURE 1:**
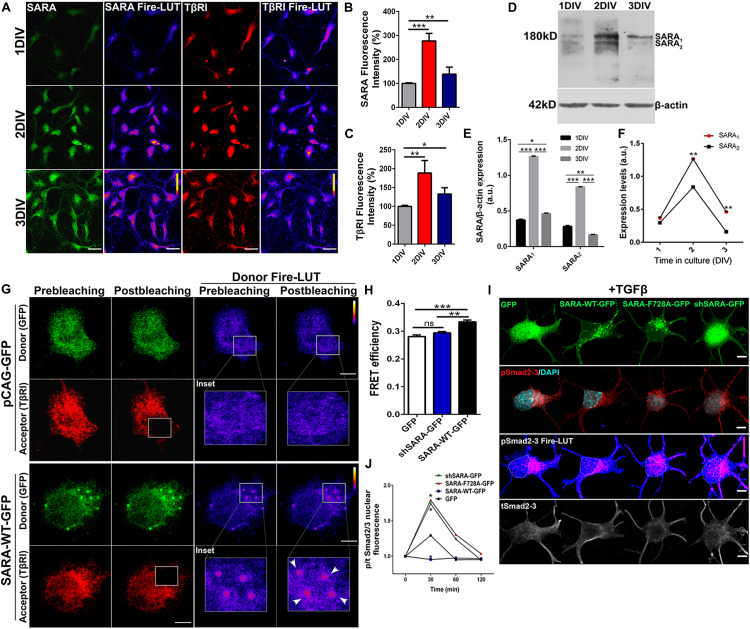
SARA controls TGFβ pathway activation in developing hippocampal neurons. **(A)** Representative images showing immunofluorescence (IF) detection of endogenous levels of SARA (green), TβRI (red) in cultured hippocampal neurons of 1, 2, and 3 DIV. Fire-LUTs are shown to clearly visualize IF levels. **(B,C)** Quantification of fluorescence intensity of SARA **(B)** and TβRI **(C)**. A total of 80 neurons were analyzed for each condition. *p < 0.05, **p < 0.005, ***p < 0.001, ANOVA, Dunnett’s post-test. **(D)** Representative detection of SARA protein levels by Western blot; protein homogenates were isolated from cultured hippocampal neurons of 1–3 DIV. **(E)** Western blotting quantification of the SARA_1_ and SARA_2_ expression normalized with the load control (β-actin). **(F)** Representation of the dynamics changes of the expression of SARA_1_ and SARA_2_ in cultured hippocampal neurons of 1–3 DIV. **(G)** Representative images showing transfected neurons with GFP or SARA-WT-GFP (green channel, donor) and immunostained for TβRI (red channel, acceptor: Alexa Fluor 546) for Acceptor Photobleaching (AP)-FRET analysis. Briefly, neurons were transfected after plating and fixed at two DIV for FRET imaging. Squares show the region bleached in the soma (magnification of these regions are shown as insets). Arrows show local interaction between SARA and TβRI at endosomes. **(H)** Quantification of FRET efficiency in control (GFP) and SARA-WT-GFP neurons at 2 DIV. **p < 0.005, ***p < 0.001 vs control (white column), ns: non-significant, ANOVA, Dunnett’s post-test (15 neurons were analyzed by condition). **(I)** Representative images of hippocampal neurons transfected with GFP, SARA-WT-GFP, shRNA-SARA, or SARA-F728A-GFP and immunostained to detect phospho-Smad2/3 (red channel), total Smad2/3 (gray channel), and DAPI levels (cyan channel). Neurons were transfected after plating and fixed at 2 DIV for IF staining. **(J)** Quantification of nuclear phospho/total Smad2/3 IF signal after TGFβ treatment (2 ng/ml) for 0, 30, 60, 120 min in 2 DIV neurons. *p < 0.05 vs control, Kruskal–Wallis test, (20 neurons were analyzed by condition). Results represent the mean of three independent neuronal cultures (*n* = 3). Scale bar: 20 μm; FRET Scale bar: 5 μm.

Previous reports have described the interaction between SARA and TβRI in non-neuronal models (Tsukazaki et al., 1998; Itoh et al., 2002); thus, we explored whether such interaction was also reproduced in neurons. To this end, we first measured endogenous levels of SARA and TβRI in cultured hippocampal neurons at 1, 2, and 3 days *in vitro* (DIV) by immunofluorescence (IF), a time-frame in which polarization takes place. Figure 1A displays representative IF of SARA and TβRI, showing that both proteins peak at 2 DIV (Figures 1B,C, Fire-LUTs are shown to clearly visualize fluorescence levels of each epitope). Previously, two isoforms of SARA have been described; SARA_1_, a high molecular weight isoform representing the full-length version, and SARA_2_, a low molecular weight isoform lacking the Smad Binding Domain (SBD) (Chang et al., 2014). To clarify which isoform is expressed in polarizing neurons, we analyzed their expression in protein homogenates isolated from 1 to 3 DIV hippocampal neurons. We found that SARA_1_ is the main variant expressed and, consistently with our IF analysis, it increases the expression at 2 DIV (Figures 1D–E). Also, the dynamics of the expression of SARA_1_ and SARA_2_ shows that SARA_1_ peaks at 2DIV compared to SARA_2_ (Figure 1F).

Then we explored the interaction between SARA and TβRI. Considering that SARA is an EE protein, we decided to analyze local interactions in EEs at neuronal soma by using *Acceptor Photobleaching Förster Resonance Energy Transfer* (AP-FRET). Therefore, hippocampal neurons were transiently transfected with plasmids encoding SARA-WT-GFP or shRNA-SARA-GFP, to knock-down SARA; an empty plasmid encoding only GFP was used as control. After 2 DIV, neurons were fixed and immunostained for TβRI detection (Figure 1G, red channel) to measure local FRET efficiency at EEs after bleaching the acceptor channel (red channel) (Figure 1G). Neurons expressing SARA-WT-GFP displayed higher FRET efficiency than controls after bleaching (Figures 1G,H, see Donor Fire-LUT), reporting an interaction between SARA and TβRI in developing neurons. On one hand, these results confirm that both SARA and TβRI are expressed within the time-frame of neuronal polarization; on the other, this data also suggests a local interaction between SARA and TβRI at EEs.

### The Loss of Function of SARA Leads to TGFβ Pathway Over-Activation in Developing Neurons

Previous reports in primary cultures of epithelial cells and cell lines showed that the ectopic expression of SARA-F728A, a mutant with dominant negative effect, induces TβRI hyperphosphorylation through a PP1c-dependent mechanism (Bennett and Alphey, 2002; Shi et al., 2004). Therefore, we explored the consequence of blocking SARA on the activation of TGFβ pathway in hippocampal neurons. As a readout of TβRI activation, we analyzed the nuclear translocation of Smad2/3 in control and SARA-inhibited neurons. For this, neurons were transfected immediately after plating with plasmids encoding GFP, SARA-WT-GFP, shRNA-SARA-GFP, or SARA-F728A-GFP. After 24 h of transfection, neurons were treated with TGFβ1 (2 ng/ml) for 0, 30, 60, and 120 min. Then, neurons were fixed and immunostained by IF to detect total and phosphorylated Smad2/3 levels (Figure 1I). We found that neurons expressing either shRNA-SARA-GFP or SARA F728A-GFP reached a peak of [phospho-Smad2/3/ total Smad2/3] nuclear fluorescence after 30 min of TGFβ1 stimulation (Figure 1J), to then decay to resting levels. However, in neurons expressing SARA-WT, and treated with TGFβ1, most of Smad2/3 remained in the cytoplasm and thereby changes in nuclear pSmad2/3 under these experimental conditions were not significant. These results suggest that over-expression of SARA-WT blocks the activation of TβRI by TGFβ1 supporting the notion that SARA is basally inhibiting the TGFβ pathway in developing neurons.

**FIGURE 2:**
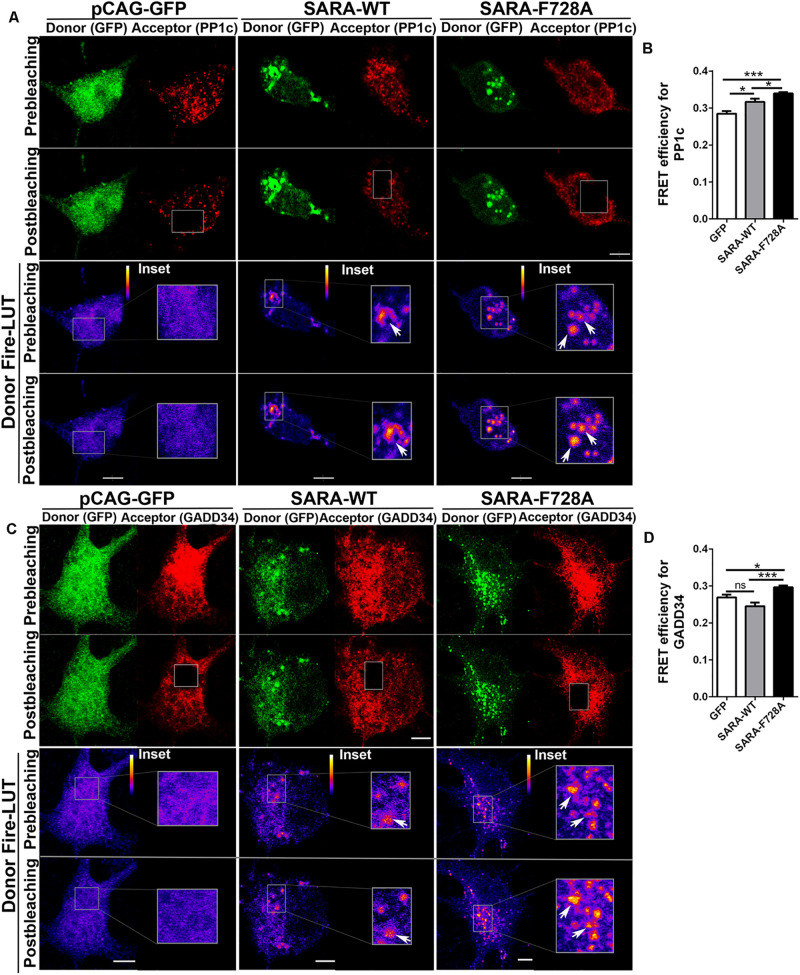
Local recruitment of PP1c and GADD34 by SARA at endosomes in developing hippocampal neurons. Neurons were transfected with GFP, SARA-WT-GFP, or SARA-F728A-GFP after plating and fixed at 2 DIV to analyze the interaction between SARA and PP1c and GADD34 at EEs by Acceptor Photobleaching-FRET. **(A,C)** Representative images of neurons transfected with GFP, SARA-WT-GFP, or SARA-F728A-GFP (green channel, donor) and immunostained for PP1c (red cannel, acceptor, **A**) or GADD34 (red channel, acceptor, **C**). Rectangles show the region bleached in the soma (magnification of these regions are shown as insets). Arrows show local interaction between SARA and TβRI at endosome. **(B,D)** Quantification of FRET efficiency for PP1c **(B)** and GADD34 **(D)**. *p < 0.05, **p < 0.005, ***p < 0.001 vs control (white column), ns: non-significant, ANOVA, Dunnett’s post-test (15 neurons were analyzed by condition). Results represent the mean of three independent cultures (*n* = 3). Scale bar: 20 μm; FRET Scale bar: 5 μm.

Previous reports in *D. melanogaster* showed that the expression of the dominant negative SARA-F728 inhibits TβRI dephosphorylation through a mechanism involving the formation of a PP1c-GADD34-Smad7 complex; therefore, endogenous SARA could be working as a membrane anchor protein to recruit PP1c, which induces TβRI dephosphorylation through a negative feedback mechanism (Shi et al., 2004). Considering this evidence, we explored whether SARA interacts with PP1c and GADD34 by AP-FRET in cultured hippocampal neurons. For this, neurons were transfected with GFP, SARA-WT-GFP or SARA-F728A-GFP immediately after plating and fixed at 2 DIV, followed by immunostaining by IF to detect endogenous PP1c (Figure 2A, red channel) or GADD34 (Figure 2C, red channel). Then, we quantified the FRET efficiency after bleaching the acceptor (red channel) in EEs located at soma for PP1c and GADD34 proteins (Figures 2B,D, respectively). As expected, FRET efficiency was higher in neurons expressing SARA-WT-GFP than controls, most likely because SARA has a binding domain for PP1c, allowing their interaction. Nevertheless, such interaction was significantly enhanced in neurons expressing the mutant SARA-F728A-GFP (Figure 2B). A similar result was obtained after analyzing the interaction between SARA and GADD34 (Figure 2D), suggesting that the SARA-F728A mutation increases the local recruitment of PP1c-GADD34, which could prevent TβRI dephosphorylation at EEs. Together, our data proposes that SARA inactivates TGFβ pathway by enhancing PP1c-GADD34 complex availability to dephosphorylate TβRI during neuronal polarization.

### Contribution of SARA to TGFβ-Dependent Polarization and Axonal Growth of Neurons in Culture

**FIGURE 3:**
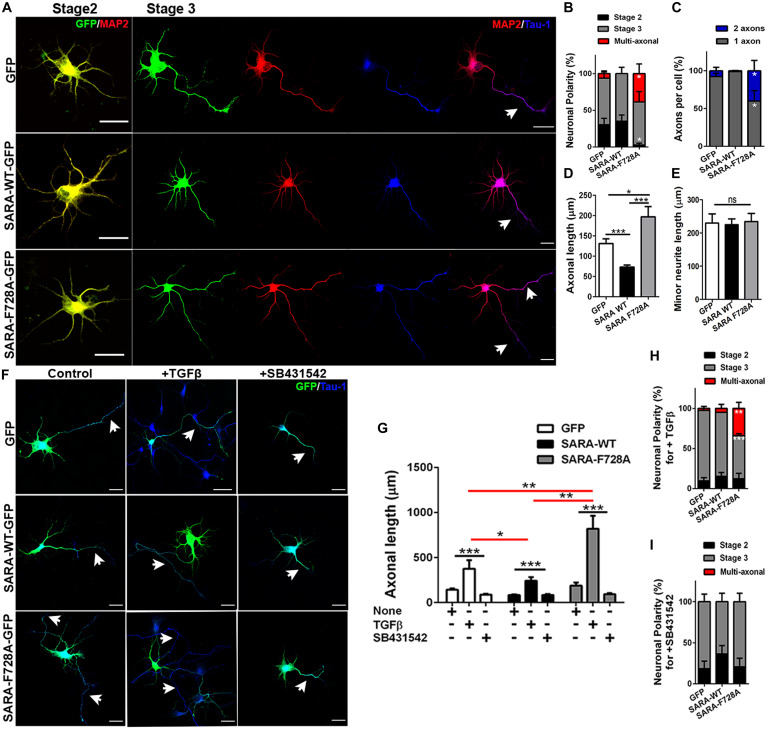
Contribution of SARA to TGFβ-dependent axonal growth during neuronal development. **(A)** Representative images of cultured hippocampal neurons transfected with GFP, SARA-WT-GFP, or SARA-F728A-GFP and immunostained to detect MAP2 (red channel) and Tau-1 (blue channel) epitopes. Briefly, neurons were transfected after plating and fixed at three DIV for IF staining. **(B–E)** Quantification of neuronal polarity acquisition (%) (stage 2: multipolar neurons; stage 3: polarized neurons and multiaxonic neurons) **(B)**, number of axons per neuron **(C)**, axonal length **(D)** and minor neurite length in 2 DIV neurons (MAP2-positive neurites 2–3 times shorter than Tau-1 positive axon) **(E)**. *p < 0.05, **p < 0.005, ***p < 0.001 vs control (white column), ns: non-significant, ANOVA, Dunnett’s post-test (45 neurons were analyzed by condition). Scale bar: 20 μm. **(F)** Representative images of 3 DIV hippocampal neurons treated with TGFβ (2 ng/ml) or SB431542 (10 μM) (TβRI inhibitor) compared with control condition and immunostained to detect Tau-1 epitope (blue). **(G)** Quantification of axonal length after TGFβ (2 ng/ml) or SB431542 (10 μM) treatments in 3 DIV neurons. **(H,I)** Quantification of neuronal polarity acquisition (%) after either TGFβ **(H)** or SB431542 treatments **(I)** in 3 DIV neurons (stage 2: multipolar neurons; stage 3: polarized neurons and multiaxonic neurons). *p < 0.05, **p < 0.005, ***p < 0.001 vs control, ns: non-significant, ANOVA, Dunnett”s post-test (30 neurons were analyzed by condition). Arrows show axons in all images. Results represent the mean of three independent cultures (*n* = 3). Scale bar: 20 μm

We next explored a functional relationship between TGFβ-mediated signaling and SARA during the first stages of polarity acquisition. Accordingly, neurons were transfected immediately after plating with plasmids encoding GFP, SARA-WT-GFP or SARA-F728A-GFP and then cells were fixed and immunostained by IF to detect MAP2 and Tau-1 epitopes at 3DIV (somato-dendritic and axonal markers, respectively) (Figure 3A). We found that neurons expressing SARA-WT-GFP showed reduced axonal growth and loss of Tau-1 distal gradient, indicating failures on axonal identity acquisition. In contrast, neurons expressing SARA-F728A-GFP showed more than one axon (defined by Tau-1 immunoreactivity, Figures 3B,C and Supplementary Figure 1). In addition, those neurons expressing SARA-F728A-GFP also exhibited longer axons, without affecting minor neurites length (Figures 3D,E), suggesting an axon-specific effect.

Then, we evaluated whether the dominant negative SARA-F728A was able to enhance the axonal growth in TβRI-inhibited neurons. Accordingly, neurons were transfected after plating with plasmids encoding GFP, SARA-WT-GFP or SARA-F728A-GFP and then, after 24 h of culture, were treated with either TGFβ1 or SB431542 (a pharmacological inhibitor of TβRI, Figure 3F and Supplementary Figure 2). As expected, the treatment with TGFβ1 increased axonal growth in control and SARA-F728A-GFP conditions; in contrast, the opposed effect was observed in SARA-WT-GFP neurons (Figures 3G,H); nevertheless, SB431542 blocked the axonal growth observed after expressing SARA-F728A-GFP (Figures 3G–I). Together, these results suggest that SARA is an endogenous inhibitor of TGFβ pathway during the first stages of neuronal polarization.

### The Over-Activation of the TGFβ Pathway Alters Both the Polarity Acquisition and Cortical Migration of Neurons *in vivo*

**FIGURE 4:**
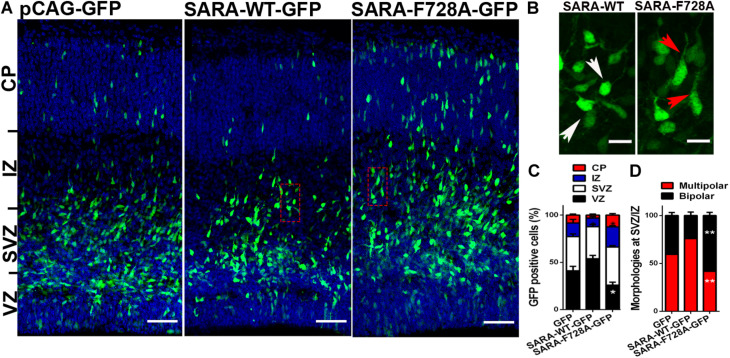
Over-activation of the TGFβ pathway by SARA-F728A-GFP ectopic expression alters both polarity acquisition and cortical migration of neurons *in vivo*. **(A)** Representative E18 coronal slices of brain cortices after *in utero* electroporation (IUE) of embryonic brains at E15, expressing pCAG-GFP, SARA-F728A-GFP, or SARA-WT-GFP and inmunostained for DAPI (blue). Three days after IUE (E18), brains were fixed and sectioned for confocal imaging. **(B)** Magnifications of insets shown in A (red squares). Neuronal morphologies and neurites developed during cortical migration are shown (red arrows: bipolar neurons; white arrows: multipolar neurons). **(C)** Quantification of GFP positive cells by layer (%). **(D)** Quantification of main morphologies detected in each condition at the boundary of SVZ/IZ. *p < 0.05 vs control, ANOVA, Dunnett’s post-test (1,500 neurons were analyzed by condition). Results represent the mean of three independent IUE experiments (*n* = 3). Scale bar: 50 μm.

Overall, our results support the hypothesis that SARA regulates TGFβ pathway during the acquisition of neuronal polarity, most likely by local recruitment of PP1c/GADD34 at EEs, affecting both polarization and axonal growth *in vitro*. Therefore, we wondered whether the SARA blockade *in vivo* would also affect the development of neurons. For this, we addressed *in utero* electroporation (IUE) experiments in embryonic (E15) mouse brains to express the following cDNA combinations: pCAG-GFP (control), pCAG-GFP + SARA-WT-GFP or pCAG-GFP + SARA-F728A-GFP. Three days after IUE (E18), we analyzed migration of cortical neurons and their morphological features (Figures 4A–D). Figure 4A shows representative images of coronal slices of cortices expressing pCAG-GFP, pCAG-GFP + SARA-WT-GFP and pCAG-GFP + SARA-F728A-GFP. We found that brain cortices expressing pCAG-GFP + SARA-F728A-GFP showed an increase in the number of cells reaching the upper zones of the cortex (IZ-CP) (compared to controls). In contrast, most of cells expressing pCAG-GFP + SARA-WT-GFP were retained at lower layers (VZ-SVZ), being unable to reach the IZ/CP within the time-frame analyzed (Figures 4A–C). Therefore, we analyzed cell morphology (multipolar or bipolar) at SVZ-IZ layers of electroporated brains (Figures 4B–D). Notably, brains expressing pCAG-GFP + SARA-F728A-GFP showed an increase in the number of bipolar phenotype (bipolar: 57%; multipolar 43%) compared to the control condition (pCAG-GFP; bipolar: 36%; multipolar: 64%, Figure 4D). Of note, most of cells expressing pCAG-GFP + SARA-WT-GFP showed a multipolar morphology (76%), a phenomenon linked with the failure on cortical radial migration exhibited by these neurons after expressing SARA-WT-GFP (Figures 4B–D). Together, these results suggest that over-activation of TGFβ pathway, achieved by the SARA blockade, accelerates the morphological transition from multipolar to bipolar neurons, affecting cortical migration of neurons to the apical layers of the cerebral cortex.

In summary, our work proposes that SARA is required to down-regulate the TGFβ pathway during the early stages of hippocampal and cortical development of neurons, most likely by the local recruitment of PP1c/GADD34 complex at EEs and dephosphorylation of TβRI. These findings support the notion that TGFβ pathway requires a proper and well-balanced modulation to promote neuronal development and function.

## Discussion

### Modulation of the TGFβ Pathway by SARA

Previous reports have shown that SARA interacts with TβRI in non-neural models. Consistently, our results confirm this hypothesis and represent the first evidence of their interaction in developing hippocampal neurons. Moreover, our AP-FRET analysis suggests that the interaction between SARA and TβRI occurs locally at EEs, the place where transduction of TGFβ signaling pathway occurs (Figure 1). These results are consistent with previous studies carried out in cell lines, reporting the interaction between SARA and TβRI using colocalization and co-immunoprecipitation assays (Tsukazaki et al., 1998; Miura et al., 2000; Itoh et al., 2002). Accordingly, SARA presents three structural motifs for biochemical interaction with proteins involved on the TGFβ pathway: (a) the SBD domain (Smad-Binding-Domain), which allows interaction with the transcriptional factors Smad2 and Smad3, (b) the PBD domain (Phosphatase-Binding-Domain), which allows SARA interaction with PP1c and, (c) the C-terminal domain, promoting docking between SARA and TβRI (Tsukazaki et al., 1998; Wu et al., 2000; Bennett and Alphey, 2002; Qin et al., 2002). In this regard, the precise contribution of SARA to the regulation of TGFβ pathway is a matter of debate. Whereas several reports associate SARA exclusively with the activation of the pathway (Wu et al., 2000; Qin et al., 2002), other reports, mainly done in *D. melanogaster* and COS-7 cells, show the opposed effect. Thus, the loss of function of SARA, by expressing the mutant SARA-F678A, turn-off the pathway by the reduction of PP1c recruitment to TβRI, leading to the hyperphosphorylation of the receptor and, consequently, the inhibition of TGFβ signaling (Bennett and Alphey, 2002; Shi et al., 2004). Accordingly, our data show that either the blockade or knock-down of SARA (by expressing SARA-F728A, the mammalian fly homolog SARA-F678A mutant, or shRNAs, respectively) increased nuclear translocation of Smad2/3. In contrast, gain of function of SARA, achieved by the ectopic expression of SARA-WT, impaired nuclear translocation of Smad2/3 (Figure 1). Collectively, these results suggest that SARA works as an inhibitor of the TGFβ pathway in basal conditions during the first stages of neuronal polarization.

Moreover, the analysis by AP-FRET showed that neurons expressing SARA-F278A had higher FRET efficiency with PP1c than SARA-WT, which occurs locally at EEs. A similar result was obtained with GADD34 (another member of the TGFβ pathway inactivation complex) (Figure 2). In this regard, neither SARA-WT nor SARA-F728A have reported binding domains for GADD34. Therefore, we hypothesize two mechanistic options. First, the F728A mutation could lead to PP1c-GADD34 mislocation. Second, F728A mutant lock the complex in a non-active state, unable to meet and dephosphorylate TβRI. Thus, our results suggest that SARA, in cooperation with several molecular partners, modulates the inactivation of TGFβ signaling in neurons. Also, considering our and previous data, the regulation of TGFβ pathway by SARA seems to be context-dependent and should be analyzed in each cell type, tissue, and developmental stage.

### Consequences of TGFβ Modulation by SARA Over Early Neuronal Development

**FIGURE 5:**
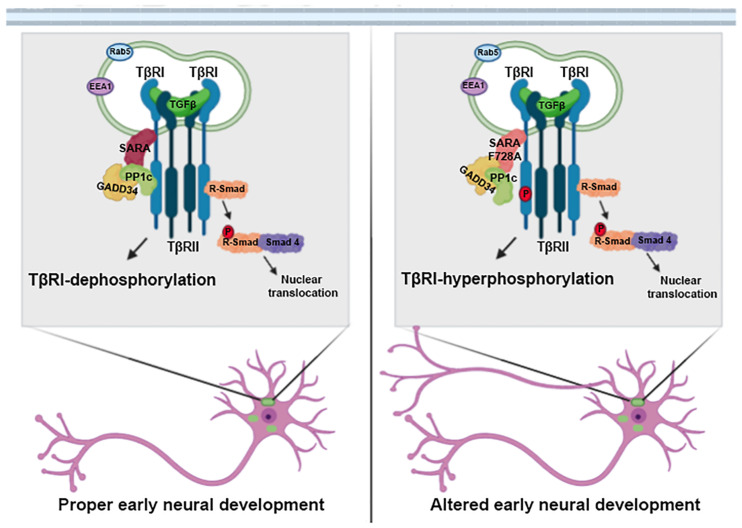
SARA is a negative regulator of the TGFβ pathway during early neuronal development. Schematic representation of the working model. SARA basally controls the deactivation of TβRI through the recruitment of the inhibitory complex PP1c/GADD34 in the endosomes of polarizing neurons. The loss of function of SARA by the dominant-negative SARA-F728A mutant, overactivated the TGFβ pathway. This functionally impacts neuronal development, promoting axonal growth, and cortical migration of neurons during brain development.

The mechanism described in this work has direct implications for neuronal development. Neurons expressing SARA-F728A have longer axons compared to their controls; and besides, these neurons displayed a multiaxonic phenotype (Figure 3 and Supplementary Figure 1). This phenotype was increased by adding TGFβ soluble or abolished when neurons were treated with a specific TGFβ receptor inhibitor (Figure 3). Similar results were obtained previously by our group, since the suppression of SARA (either in knock-down or knock-out neurons) leads to the formation of supernumerary axons, suggesting a direct effect on polarity acquisition (Arias et al., 2015). Moreover, exogenous TGFβ is able to drive axonal growth and differentiation, and over-expression of the TβRI leads to multiaxonic neurons (Yi et al., 2010). Therefore, our work extends the knowledge regarding the regulation of TGFβ signaling, reporting a functional cross-talk between SARA and TGFβ needed for axonal growth and polarization of hippocampal neurons (Figure 5).

During corticogenesis, newborn neurons exhibit multipolar morphologies with a symmetric arrangement of immature neurites. However, cortical neurons replaced these neurites after axon specification to develop a bipolar morphology able to migrate toward the pial surface (Ehlers and Polleux, 2010; Kawauchi, 2015). Then, when radial migration takes place, most of the neurons located at SVZ-IZ layers exhibit a bipolar morphology, which is required for proper cortical migration (Kriegstein and Noctor, 2004; Hatanaka and Yamauchi, 2013; Mestres et al., 2016; Wilson et al., 2020). Embryonic brains expressing SARA-F728A presented a higher number of neurons with bipolar phenotype, as well as an increase in the number of neurons reaching the upper layers of the cortex, suggesting an enhancement of cortical migration (Figure 4). However, the impact on wiring and connectivity required to be examined. Based on *in vitro* and *in vivo* studies, the gain of function of TGFβ signaling, after blocking SARA, alters the balance of polarity acquisition, evidenced by the development of multiaxonic neurons, axonal overgrowth, and improvements on cortical migration of neurons (Figure 5). Considering our data, these phenotypes probably rely on TβRI hyperphosphorylation and amplification of TGFβ signaling, being consistent with *ex vivo* results of rat brains where treatment with TGFβ1 accelerates the migration of newborn neurons (Siegenthaler and Miller, 2004). Moreover, TGFβ1/2/3 promote neuritic growth of chicken DRG explants (Unsicker et al., 1996); consistently, a similar effect was observed in axons of rat hippocampal neurons, without affecting dendritogenesis (Ishihara et al., 1994). In addition, inhibition of TGFβ signaling, by reducing Smad4 levels during embryogenesis, is required for the proper spatio-temporal development of granular progenitor cells (Fernandes et al., 2012).

The knock-down of SARA *in vivo* (by IUE) impairs cortical development of mouse brains, reducing cortical migration of neurons even in postnatal days. In this context, our group previously showed an abnormal supply of the L1 cell adhesion molecule to growing axons, with a strong delay in neuronal development. Moreover, the suppression of SARA alters leading process orientation and multipolar-bipolar transition through an L1-dependent mechanism (Mestres et al., 2016). Conversely, the expression of SARA-F728A, did not arrested neurons at the IZ during the early stages of the neocortex development, suggesting that the PP1c domain is not involved in the normal localization of L1. The fact that the F728A mutation increases the number of bipolar neurons, most likely due to increased phosphorylation of TβRI suggest that different domains in SARA may control different aspects of neuronal differentiation mediated by TGFβ dependent and independent mechanisms. However, the control of L1 expression by TGFβ has not been described yet, thus it may be important in further studies to address whether TGFβ and L1 functions are mechanistically connected. Nevertheless, TGFβ1 is able to up-regulate the expression of cell-adhesion molecules, like N-CAM, integrin α3, αv, and β1 (Siegenthaler and Miller, 2004). In contrast, the physiological relevance of suppressing SARA is not necessarily equivalent to its loss of function (e.g., by SARA mutant expression), at least in the context of TGFβ signaling pathway. In this regard, SARA knock-down decreases its protein levels, while SARA-F728A mutant preserves a non-functional PP1c domain, making both strategies not exactly equivalent. Together, these results suggest that TGFβ over-activation by blocking SARA alters polarization and cortical migration *in vivo*.

Finally, the signaling of TGFβ controls a wide variety of physiological and pathological processes, including neural differentiation at embryonic development (Tomoda et al., 1996; Unsicker et al., 1996; Yi et al., 2010), inflammatory and immune response (Gorelik et al., 2002; Takimoto et al., 2010), axonal regeneration after injury (Sulaiman, 2016; Carmichael et al., 2017), and the progression of several pathologies, including Alzheimer’s and Parkinson’s diseases (Vawter et al., 1996; Zhang et al., 2016; Caraci et al., 2018), psychiatric disorders (Benes et al., 2007; Sun et al., 2010), and carcinogenesis (Mao et al., 2006; Benes et al., 2007; Sun et al., 2010; David and Massague, 2018; Hachim et al., 2018; Chen et al., 2020), among others. Although several reports have described in detail the regulation of TGFβ pathway (Wrana et al., 1994; Massague, 1998; Massague and Chen, 2000), our work stress the action of non-canonical regulators like SARA, a protein usually associated with endosomes and trafficking machinery. Previous studies have described that the trafficking of SARA endosomes plays a crucial role during the development of *D. melanogaster* and *D. rerio*, determining the fate of neuronal precursors during cell division (Loubery et al., 2014, 2017; Derivery et al., 2015; Kressmann et al., 2015). In addition to trafficking functions, these works suggest that SARA endosomes are also related to the regulation of cell signaling, such as the Notch/Delta pathway (Coumailleau et al., 2009; Loubery et al., 2014; Kressmann et al., 2015). Overall, it seems that SARA is required not only to carry out trafficking functions, such as endosome segregation but also for proper modulation of molecules and signaling pathways required for neuronal development and growth.

In conclusion, the molecular mechanism described in this work suggest that SARA is an endogenous negative regulator able to fine-tune the TGFβ signaling pathway to achieve polarization and axonal growth of neurons properly.

## Materials and Methods

### Primary Culture of Hippocampal Neurons From Rat Embryonic Brains

Cultures of embryonic hippocampal neurons were done following the protocol of Kaech and Banker (2006). Briefly, pregnant Wistar rats (E18.5) were sacrificed and embryos were removed to isolate the hippocampus, followed by enzymatical and mechanical digestion. Neurons were plated in multi-well dishes containing glass coverslips previously treated with 1 mg/ml of poly-L-lysine (Sigma-Aldrich, St. Louis, Missouri, United States) using Dulbeco’s Modified Eagles Medium (DMEM; Thermo Fisher Scientific, Massachusetts, United States), supplemented with 10% horse serum (Gibco, Thermo Fisher Scientific, Massachusetts, United States), for 1 h at 37°C and 5% CO_2_. Then, plating medium was replaced by Neurobasal supplemented with B27, Glutamax, Sodium Pyruvate and antibiotics (Pen/Strep) (Thermo Fisher Scientific, Massachusetts, United States). All of the experiments were approved by the Bioethical Research Committee of INIMEC-CONICET-UNC and conducted following the guidelines of the manual for animal experimentation approved by the institutional animal care committee (INIMEC-National Research Council and Universidad Nacional de Córdoba, Argentina), the National Department of Animal Care and Health (SENASA-Argentina) and Approval to conduct the study was granted by the Animal Care and Ethics Committee (CICUAL) of INIMEC-CONICET-UNC (Resolution numbers 006/2017A and 012/2017A). Efforts were made to minimize animal suffering and to reduce the number of animals used.

### Transfection of cDNA Coding Vectors

Constructs pCAG-GFP, pRK5-SARA-WT-GFP, and pCAG-shRNA-SARA were kindly provided by Dr. Ching-Hwa Sung (Weill Cornell Medical College, United States). The target sequence of the shSARA is: 5′-AGCTTAAAAGGGAGAAC ATGATGAGTGCCTCCATGGAGGCACTCATCATGTTCTCC-3′. This construction was designed and developed by Dr. Jen-Zen Chuang (Chuang et al., 2007) and used in Mestres et al., 2016. The shRNA sequence targets both SARA isoforms since it is within the PBD domain. pRK5-SARA-F728A-GFP was designed by our group from pRK5-SARA-WT-GFP using a Quick Change Site-Directed Mutagenesis Kit (Stratagene, California, United States) with the following primers: Forward: 5′-G CAGAGGCGAGTTTGGGCTGCTGCTGATGGGATCTTGCC-3′ and Reverse: 5′-GGCAAGATCCCATCAGCAGCCCAAAC TCGCCTCTGC-3′. Then pRK5-SARA-F728A-GFP was amplified in DH5a cells and colonies (Amp-resistant) were selected for DNA purification using a mini-purification kit (Promega, Wisconsin, United States). Purified pRK5-SARA-F728A-GFP was sequenced in Macrogen (Korea) to verify correct site-directed mutation. Subsequently, the vector was again amplified in DH5a cells and colonies were selected. Amp-resistant bacteria were selected for DNA purification (Qiagen, Hilden, Germany).

Neurons were plated in 10% HS MEM medium for 1 h, when the medium was replaced, were transiently transfected with Lipofectamine 2000 (Life Technologies, CA, United States), following manufacturer’s instructions. Complexes containing pCAG-GFP, SARA-WT-GFP, SARA-F728A-GFP, or shRNA-SARA plus Lipofectamine 2000 were resuspended in Optimem (Thermo Fisher Scientific, Massachusetts, United States) and dissolved in Neurobasal for transfection; after 2 h it was replaced by Neurobasal supplemented with B27, Glutamax, Sodium Pyruvate and antibiotics (Thermo Fisher Scientific, Massachusetts, United States). Experiments were performed at 18, 48, or 72 h after cDNA transfection.

### Primary Antibodies

The following antibodies were used in this study: anti-SARA [mouse, sc-133071; 1:200 for immunoblotting and 1:100 for immunofluorescence (IF)], anti-PP1c (mouse, sc-7482; 1:50 for IF), anti-GADD34 (mouse, sc-373815; 1:50 for IF), anti-TβRI (mouse, sc-101574; 1:100 for IF) and anti-Smad2/3 (mouse, sc-398844; 1:100 for IF); all these antibodies were purchased from Santa Cruz Biotechnology (Dallas, Texas, United States). The antibody anti-MAP2 (rabbit, 1:500) and anti-Tau-1 (mouse, 1:500) were from Merck Millipore (Darmstadt, Germany). Antibody anti-pSamd2/3 (rabbit, s465/s467, E8F3R, 1:50 for IF) was from Cell Signaling (Danvers, MA, United States) and anti-phospho TβRI (rabbit, ser165, Lot: DY1241; 1:50 for IF) was acquired from Elabscience (Houston, Texas, United Sates).

### TGFβ and SB431542 Treatment

Neurons were also treated with TGFβ1 (Lot. 0713354 D1014 from Acris GmbH, Germany) [physiological concentration: 2 ng/ml, (Fogel-Petrovic et al., 2007)] or SB431542 (10 μM) (DaCosta Byfield et al., 2004; Koo et al., 2015) (Lot. N° 0504746-44, Cayman Chemical Company, Michigan, United States); both TGFβ1 and SB431542 were added 18 h after plating.

### *In utero* Electroporation (IUE) and Imaging Acquisition

IUE were done following previous reports (Niwa et al., 1991; Saito and Nakatsuji, 2001). Briefly, pregnant E15.5 C57bl/6 mice were anesthetized with isoflurane/oxygen mix (4% for induction and 2% for maintenance) during the whole surgery, using Tramadol (5 mg/kg) as analgesia during the procedure. Embryos were exposed out of the maternal belly for local injection of pCAG-GFP, pCAG-GFP + SARA-WT-GFP or pCAG-GFP + SARA-F728A-GFP into lateral ventricle of the brain. Fast green FCF dye (Sigma-Aldrich, St. Louis, Missouri, United States; catalog number F7252) was co-injected with DNAs to visualize injections. Then, brains were electroporated using a BTX electroporator (ECM 830 Square Wave Pulse generator, Fisher scientific, MA, United States) (Δ*V* = 39 V; pulses: 5; duration: 50 ms; intervals between pulses: 950 ms) with Tweezers w/Variable Gap 2 Square Platinum Electrodes (Nepagene, Japan, CUY647P2 × 2). At E18, embryos were sacrificed to check GFP expression in control and SARA-WT or SARA-F728A genetic contexts. Brains expressing GFP were fixed and immersed into a 30% w/v sucrose solution for 24 h, at 4°C. Cerebral cortex of GFP-positive brains was sliced into 50 μm cortical sections using a cryostat (Leica CM 1850, Leica Biosystems, Illinois, United States). Before the imaging, tissues were permeabilized with 0.3% v/v Triton x-100-PBS solution, followed by DAPI staining (15 min at RT). Samples were mounted in Mowiol solution (Sigma-Aldrich, St. Louis, Missouri, United States) imaging in a Zeiss LSM-800 confocal microscopy (Zeiss, Oberkochen, Germany). Images were acquired with a 20× air objective. Several fields were acquired (from the ventricular zone to the cortical plate). For migration analysis, the embryonic neocortex was divided in 4 zones (from the bottom to the top): ventricular zone (VZ), subventricular zone (SVZ), intermediate zone (IZ) and cortical plate (CP) (Fuentes et al., 2012).

### Measurement of Acceptor *Photobleaching Förster Resonance Energy Transfer* (FRET)

Neurons were cultured in p24 multi-wells (5 × 10^4^ cells/well) and transfected with cDNA–coding vectors as detailed before. Immunofluorescence detections were done for TβRI, PP1c, or GADD34. FRET efficiency measurements were performed as described previously (Grzanka et al., 2014; Bignante et al., 2018). De-quenching of the donor (GFP) after selective photo-bleaching of the acceptor causes an increase in donor emission (460–490 nm) that was quantified. For this, pre and post-bleaching images of the donor and acceptor were obtained. The measurement of FRET efficiency was carried out by selecting the photo boiled region of the acceptor and measuring FRET eff = 1-Pre-Donor/Post-Donor. ROIs were selected in areas with endosome overexpression or randomized in the control conditions.

### Measurement of Smad2/3 Nuclear Translocation

Transfected neurons were treated with TGFβ1 (2 ng/ml) at 18 h of culture for 0, 30, 60, and 120 min. Then, neurons were fixed and immunostained to detect total Smad2/3 and phospho-Smad2/3; DAPI was used to stain the nuclei. The ratio [phospho-Smad2/3/t Smad2/3] was quantified in the nuclei in a ROI defined by DAPI staining (Ranganathan et al., 2007). The result was obtained from a Z projection of a stack of confocal sections acquired by Zeiss LSM-800 confocal microscopy (Zeiss, Oberkochen, Germany).

### Image Acquisition, Analysis, and Statistics

Cells were visualized using either a spectral (Olympus Fluoview 1000, Shinjuku City, Tokyo, Japan) or LSM-800 (Zeiss, Oberkochen, Germany) (Lasers: 488, 533, and 633; resolution *X* = 1024; *Y* = 1024, and *Z* = 0.3–0.5 μm; Objectives: 63×: Plan-Apochromat 63×/1.40 Oil DICM27 and 20×: Objective 20× LD Apochromat 20×/0.40, both inverted confocal microscopes. Post-imaging analysis were done using Fiji-ImageJ (NIH, United States) (Schindelin et al., 2012). Results represent the mean ± SEM from of at least three independent cultures (*n* = 3). The number of neurons analyzed by each experiment are indicated in the figure legends. Shapiro–Wilk normalcy test was used to evaluate normal distribution of datasets. Student’s *t-*tests or ANOVA were performed for parametric data. Mann–Whitney or Kruskal–Wallis tests were used for non-parametric data using GraphPad Prism 5.

## Data Availability Statement

The raw data supporting the conclusions of this article will be made available by the authors, without undue reservation.

## Ethics Statement

The animal study was reviewed and approved by the Animal Care and Ethics Committee (CICUAL) of INIMEC-CONICET-UNC.

## Author Contributions

VR-S, CW, CO, CG-B, and CC designed the research and wrote and edited the manuscript. VR-S performed the research. VR-S, CW, CG-B, and CC analyzed the data. All authors contributed to the article and approved the submitted version.

## Conflict of Interest

The authors declare that the research was conducted in the absence of any commercial or financial relationships that could be construed as a potential conflict of interest.

## Supplementary Material

FIGURE S1 Over-activation of the TGFβ pathway by SARA-F728A-GFP generates multi-axonal neurons. **(A)** Representative images of cultured hippocampal neurons transfected with SARA-F728A-GFP and immunostained to detect MAP2 (red channel) and Tau-1 (blue channel) epitopes. Neurons were transfected after plating and fixed at 3 DIV for IF staining. Arrows show axons in the images. Results represent the mean of three independent cultures (*n* = 3). Scale bar: 20 μm.

FIGURE S2 Contribution of SARA to TGFβ-dependent axonal growth during neuronal development. **(A)** Representative images of three DIV hippocampal neurons transfected with GFP, SARA-WT-GFP, or SARA-F728A-GFP and treated with TGFβ (2 ng/ml) or SB431542 (10 μM) (TβRI inhibitor). Results represent different examples from three independent cultures (*n* = 3). Scale bar: 20 μm.

## References

[B1] Akhurst R. J. Hata A. (2012). Targeting the TGFbeta signalling pathway in disease. *Nat. Rev. Drug Discov.* 11 790–811. 10.1038/nrd3810 23000686 PMC3520610

[B2] Arias C. I. Siri S. O. Conde C. (2015). Involvement of SARA in axon and dendrite growth. *PLoS One* 10:e0138792. 10.1371/journal.pone.0138792 26405814 PMC4583221

[B3] Arimura N. Kaibuchi K. (2007). Neuronal polarity: from extracellular signals to intracellular mechanisms. *Nat. Rev. Neurosci.* 8 194–205. 10.1038/nrn2056 17311006

[B4] Attisano L. Wrana J. L. (2002). Signal transduction by the TGF-beta superfamily. *Science* 296 1646–1647. 10.1126/science.1071809 12040180

[B5] Benes F. M. Lim B. Matzilevich D. Walsh J. P. Subburaju S. Minns M. (2007). Regulation of the GABA cell phenotype in hippocampus of schizophrenics and bipolars. *Proc. Natl. Acad. Sci. U.S.A.* 104 10164–10169. 10.1073/pnas.0703806104 17553960 PMC1888575

[B6] Bignante E. A. Ponce N. E. Heredia F. Musso J. Krawczyk M. C. Millan J. (2018) APP/Go protein Gβγ-complex signaling mediates Aβ degeneration and cognitive impairment in Alzheimer’s disease models. *Neurobiol. Aging* 64, 44–57. 10.1016/j.neurobiolaging.2017.12.013 29331876

[B7] Bennett D. Alphey L. (2002). PP1 binds Sara and negatively regulates Dpp signaling in *Drosophila melanogaster*. *Nat. Genet.* 31 419–423. 10.1038/ng938 12134149

[B8] Caceres A. Ye B. Dotti C. G. (2012). Neuronal polarity: demarcation, growth and commitment. *Curr. Opin. Cell Biol.* 24 547–553. 10.1016/j.ceb.2012.05.011 22726583 PMC3425660

[B9] Caraci F. Spampinato S. F. Morgese M. G. Tascedda F. Salluzzo M. G. Giambirtone M. C. (2018). Neurobiological links between depression and AD: The role of TGF-beta1 signaling as a new pharmacological target. *Pharmacol. Res.* 130 374–384. 10.1016/j.phrs.2018.02.007 29438781

[B10] Carmichael S. T. Kathirvelu B. Schweppe C. A. Nie E. H. (2017). Molecular, cellular and functional events in axonal sprouting after stroke. *Exp. Neurol.* 287(Pt 3), 384–394. 10.1016/j.expneurol.2016.02.007 26874223 PMC4980303

[B11] Chang H. M. Lin Y. Y. Tsai P. C. Liang C. T. Yan Y. T. (2014). The FYVE domain of Smad anchor for receptor activation (SARA) is required to prevent skin carcinogenesis, but not in mouse development. *PLoS One* 9:e105299. 10.1371/journal.pone.0105299 25170969 PMC4149420

[B12] Chen Y. Di C. Zhang X. Wang J. Wang F. Yan J. F. (2020). Transforming growth factor beta signaling pathway: a promising therapeutic target for cancer. *J. Cell Physiol.* 235 1903–1914. 31332789 10.1002/jcp.29108

[B13] Chuang J. Z. Yeh T. Y. Bollati F. Conde C. Canavosio F. Caceres A. (2005). The dynein light chain Tctex-1 has a dynein-independent role in actin remodeling during neurite outgrowth. *Dev. Cell.* 9 75–86. 10.1016/j.devcel.2005.04.003 15992542 PMC3857739

[B14] Chuang J. Z. Zhao Y. Sung C. H. (2007). SARA-regulated vesicular targeting underlies formation of the light-sensing organelle in mammalian rods. *Cell* 130 535–547. 10.1016/j.cell.2007.06.030 17693260 PMC3857750

[B15] Cid-Arregui A. De Hoop M. Dotti C. G. (1995). Mechanisms of neuronal polarity. *Neurobiol. Aging* 16 239–243. 10.1016/0197-4580(94)00190-c 7566334

[B16] Conde C. Caceres A. (2009). Microtubule assembly, organization and dynamics in axons and dendrites. *Nat. Rev. Neurosci.* 10 319–332. 10.1038/nrn2631 19377501

[B17] Coumailleau F. Furthauer M. Knoblich J. A. Gonzalez-Gaitan M. (2009). Directional Delta and Notch trafficking in Sara endosomes during asymmetric cell division. *Nature* 458 1051–1055. 10.1038/nature07854 19295516

[B18] DaCosta Byfield S. Major C. Laping N. J. Roberts A. B. (2004). SB-505124 is a selective inhibitor of transforming growth factor-beta type I receptors ALK4. ALK5 and ALK7. *Mol. Pharmacol.* 65 744–752. 10.1124/mol.65.3.744 14978253

[B19] David C. J. Massague J. (2018). Contextual determinants of TGFbeta action in development, immunity and cancer. *Nat. Rev. Mol. Cell Biol.* 19 419–435. 10.1038/s41580-018-0007-0 29643418 PMC7457231

[B20] Derivery E. Seum C. Daeden A. Loubery S. Holtzer L. Julicher F. (2015). Polarized endosome dynamics by spindle asymmetry during asymmetric cell division. *Nature* 528 280–285. 10.1038/nature16443 26659188

[B21] Dotti C. G. Sullivan C. A. Banker G. A. (1988). The establishment of polarity by hippocampal neurons in culture. *J. Neurosci.* 8 1454–1468. 10.1523/jneurosci.08-04-01454.1988 3282038 PMC6569279

[B22] Ehlers M. D. Polleux F. (2010). Neuronal and glial cell biology. *Curr. Opin. Neurobiol.* 20 529–530. 20678922 10.1016/j.conb.2010.06.004

[B23] Falk J. Konopacki F. A. Zivraj K. H. Holt C. E. (2014). Rab5 and Rab4 regulate axon elongation in the *Xenopus* visual system. *J. Neurosci.* 34 373–391. 10.1523/jneurosci.0876-13.2014 24403139 PMC3870927

[B24] Fernandes M. Antoine M. Hebert J. M. (2012). SMAD4 is essential for generating subtypes of neurons during cerebellar development. *Dev. Biol.* 365 82–90. 10.1016/j.ydbio.2012.02.017 22370000 PMC3322275

[B25] Fogel-Petrovic M. Long J. A. Misso N. L. Foster P. S. Bhoola K. D. Thompson P. J. (2007). Physiological concentrations of transforming growth factor beta1 selectively inhibit human dendritic cell function. *Int. Immunopharmacol.* 7 1924–1933. 10.1016/j.intimp.2007.07.003 18039529

[B26] Fuentes P. Canovas J. Berndt F. A. Noctor S. C. Kukuljan M. (2012). CoREST/LSD1 control the development of pyramidal cortical neurons. *Cereb. Cortex* 22 1431–1441. 10.1093/cercor/bhr218 21878487

[B27] Funahashi Y. Watanabe T. Kaibuchi K. (2020). Advances in defining signaling networks for the establishment of neuronal polarity. *Curr. Opin. Cell Biol.* 63 76–87. 10.1016/j.ceb.2019.12.009 32018218

[B28] Gorelik L. Constant S. Flavell R. A. (2002). Mechanism of transforming growth factor beta-induced inhibition of T helper type 1 differentiation. *J. Exp. Med.* 195 1499–1505. 10.1084/jem.20012076 12045248 PMC2193549

[B29] Grzanka D. Gagat M. Izdebska M. (2014). Involvement of the SATB1/F-actin complex in chromatin reorganization during active cell death. *Int. J. Mol. Med.* 33, 1441–1450. 10.3892/ijmm.2014.1710 24676287 PMC4055304

[B30] Hachim M. Y. Hachim I. Y. Dai M. Ali S. Lebrun J. J. (2018). Differential expression of TGFbeta isoforms in breast cancer highlights different roles during breast cancer progression. *Tumour. Biol.* 40:1010428317748254. 10.1177/1010428317748254 29320969

[B31] Hatanaka Y. Yamauchi K. (2013). Excitatory cortical neurons with multipolar shape establish neuronal polarity by forming a tangentially oriented axon in the intermediate zone. *Cereb. Cortex* 23 105–113. 10.1093/cercor/bhr383 22267309

[B32] Hu Y. Chuang J. Z. Xu K. McGraw T. G. Sung C. H. (2002). SARA, a FYVE domain protein, affects Rab5-mediated endocytosis. *J. Cell Sci.* 115(Pt 24), 4755–4763. 10.1242/jcs.00177 12432064 PMC3899687

[B33] Ishihara A. Saito H. Abe K. (1994). Transforming growth factor-beta 1 and -beta 2 promote neurite sprouting and elongation of cultured rat hippocampal neurons. *Brain Res.* 639 21–25. 10.1016/0006-8993(94)91759-0 8180834

[B34] Itoh F. Divecha N. Brocks L. Oomen L. Janssen H. Calafat J. (2002). The FYVE domain in Smad anchor for receptor activation (SARA) is sufficient for localization of SARA in early endosomes and regulates TGF-beta/Smad signalling. *Genes Cells* 7 321–331. 10.1046/j.1365-2443.2002.00519.x 11918675

[B35] Kaech S. Banker G. (2006). Culturing hippocampal neurons. *Nat. Protoc.* 1 2406–2415. 10.1038/nprot.2006.356 17406484

[B36] Kawauchi T. (2015). Cellullar insights into cerebral cortical development: focusing on the locomotion mode of neuronal migration. *Front. Cell Neurosci.* 9:394. 10.3389/fncel.2015.00394 26500496 PMC4595654

[B37] Koo B. H. Kim Y. Cho Y. J. Kim D. S. (2015). Distinct roles of transforming growth factor-beta signaling and transforming growth factor-beta receptor inhibitor SB431542 in the regulation of p21 expression. *Eur. J. Pharmacol.* 764 413–423. 10.1016/j.ejphar.2015.07.032 26187313

[B38] Kostaras E. Pedersen N. M. Stenmark H. Fotsis T. Murphy C. (2014). SARA and RNF11 at the crossroads of EGFR signaling and trafficking. *Methods Enzymol.* 535 225–247. 10.1016/b978-0-12-397925-4.00014-6 24377927

[B39] Kostaras E. Sflomos G. Pedersen N. M. Stenmark H. Fotsis T. Murphy C. (2013). SARA and RNF11 interact with each other and ESCRT-0 core proteins and regulate degradative EGFR trafficking. *Oncogene* 32 5220–5232. 10.1038/onc.2012.554 23222715

[B40] Kressmann S. Campos C. Castanon I. Furthauer M. Gonzalez-Gaitan M. (2015). Directional Notch trafficking in Sara endosomes during asymmetric cell division in the spinal cord. *Nat. Cell Biol.* 17 333–339. 10.1038/ncb3119 25706234

[B41] Kriegstein A. R. Noctor S. C. (2004). Patterns of neuronal migration in the embryonic cortex. *Trends Neurosci.* 27 392–399. 10.1016/j.tins.2004.05.001 15219738

[B42] Loubery S. Daeden A. Seum C. Holtzer L. Moraleda A. Damond N. (2017). Sara phosphorylation state controls the dispatch of endosomes from the central spindle during asymmetric division. *Nat. Commun.* 8:15285. 10.1038/ncomms15285 PMC5467175 28585564

[B43] Loubery S. Seum C. Moraleda A. Daeden A. Furthauer M. Gonzalez-Gaitan M. (2014). Uninflatable and Notch control the targeting of Sara endosomes during asymmetric division. *Curr. Biol.* 24 2142–2148. 10.1016/j.cub.2014.07.054 25155514

[B44] Mao J. H. Saunier E. F. de Koning J. P. McKinnon M. M. Higgins M. N. Nicklas K. (2006). Genetic variants of Tgfb1 act as context-dependent modifiers of mouse skin tumor susceptibility. *Proc. Natl. Acad. Sci. U.S.A.* 103 8125–8130. 10.1073/pnas.0602581103 16702541 PMC1472440

[B45] Massague J. (1998). TGF-beta signal transduction. *Annu. Rev. Biochem.* 67 753–791. 9759503 10.1146/annurev.biochem.67.1.753

[B46] Massague J. Chen Y. G. (2000). Controlling TGF-beta signaling. *Genes Dev.* 14 627–644. 10733523

[B47] Mestres I. Chuang J. Z. Calegari F. Conde C. Sung C. H. (2016). SARA regulates neuronal migration during neocortical development through L1 trafficking. *Development* 143 3143–3153. 10.1242/dev.129338 27471254 PMC5047672

[B48] Meyers E. A. Kessler J. A. (2017). TGF-beta family signaling in neural and neuronal differentiation, development, and function. *Cold Spring Harb. Perspect. Biol.* 9:a022244. 10.1101/cshperspect.a022244 28130363 PMC5538418

[B49] Miura S. Takeshita T. Asao H. Kimura Y. Murata K. Sasaki Y. (2000). Hgs (Hrs), a FYVE domain protein, is involved in Smad signaling through cooperation with SARA. *Mol. Cell Biol.* 20 9346–9355. 10.1128/mcb.20.24.9346-9355.2000 11094085 PMC102191

[B50] Mogi M. Harada M. Kondo T. Narabayashi H. Riederer P. Nagatsu T. (1995). Transforming growth factor-beta 1 levels are elevated in the striatum and in ventricular cerebrospinal fluid in Parkinsons disease. *Neurosci. Lett.* 193 129–132. 10.1016/0304-3940(95)11686-q 7478158

[B51] Niwa H. Yamamura K. Miyazaki J. (1991). Efficient selection for high-expression transfectants with a novel eukaryotic vector. *Gene* 108 193–199. 10.1016/0378-1119(91)90434-d 1660837

[B52] Panopoulou E. Gillooly D. J. Wrana J. L. Zerial M. Stenmark H. Murphy C. (2002). Early endosomal regulation of Smad-dependent signaling in endothelial cells. *J. Biol. Chem.* 277 18046–18052. 10.1074/jbc.m107983200 11877415

[B53] Qin B. Y. Lam S. S. Correia J. J. Lin K. (2002). Smad3 allostery links TGF-beta receptor kinase activation to transcriptional control. *Genes Dev.* 16 1950–1963. 10.1101/gad.1002002 12154125 PMC186427

[B54] Ranganathan P. Agrawal A. Bhushan R. Chavalmane A. K. Kalathur R. K. Takahashi T. (2007). Expression profiling of genes regulated by TGF-beta: differential regulation in normal and tumour cells. *BMC Genomics* 8:98. 10.1186/1471-2164-8-98 17425807 PMC1858692

[B55] Rozes-Salvador V. Siri S. O. Musri M. M. Conde C. (2018). New player in endosomal trafficking: differential roles of smad anchor for receptor activation (SARA) protein. *Mol. Cell Biol.* 38:e00446-18. 10.1128/MCB.00446-18 PMC6275186 30275343

[B56] Saito T. Nakatsuji N. (2001). Efficient gene transfer into the embryonic mouse brain using in vivo electroporation. *Dev. Biol.* 240 237–246. 10.1006/dbio.2001.0439 11784059

[B57] Sann S. Wang Z. Brown H. Jin Y. (2009). Roles of endosomal trafficking in neurite outgrowth and guidance. *Trends Cell Biol.* 19 317–324. 10.1016/j.tcb.2009.05.001 19540123

[B58] Schindelin J. Arganda-Carreras I. Frise E. Kaynig V. Longair M. Pietzsch T. (2012). Fiji: an open-source platform for biological-image analysis. *Nat. Methods* 9 676–682. 10.1038/nmeth.2019 22743772 PMC3855844

[B59] Shi W. Sun C. He B. Xiong W. Shi X. Yao D. (2004). GADD34-PP1c recruited by Smad7 dephosphorylates TGFbeta type I receptor. *J. Cell Biol.* 164 291–300. 10.1083/jcb.200307151 14718519 PMC2172339

[B60] Shi Y. Massague J. (2003). Mechanisms of TGF-beta signaling from cell membrane to the nucleus. *Cell* 113 685–700. 10.1016/s0092-8674(03)00432-x 12809600

[B61] Siegenthaler J. A. Miller M. W. (2004). Transforming growth factor beta1 modulates cell migration in rat cortex: effects of ethanol. *Cereb. Cortex* 14 791–802. 10.1093/cercor/bhh039 15084492

[B62] Sulaiman W. A. (2016). Transforming growth factor-beta promotes axonal regeneration after chronic nerve injury. *Spine* 41(Suppl. 7):S29. 10.1097/BRS.0000000000001435 27015069

[B63] Sun M. Gewirtz J. C. Bofenkamp L. Wickham R. J. Ge H. OConnor M. B. (2010). Canonical TGF-beta signaling is required for the balance of excitatory/inhibitory transmission within the hippocampus and prepulse inhibition of acoustic startle. *J. Neurosci.* 30 6025–6035. 10.1523/jneurosci.0789-10.2010 20427661 PMC6632596

[B64] Takano T. Urushibara T. Yoshioka N. Saito T. Fukuda M. Tomomura M. (2014). LMTK1 regulates dendritic formation by regulating movement of Rab11A-positive endosomes. *Mol. Biol. Cell* 25 1755–1768. 10.1091/mbc.e14-01-0675 24672056 PMC4038502

[B65] Takimoto T. Wakabayashi Y. Sekiya T. Inoue N. Morita R. Ichiyama K. (2010). Smad2 and Smad3 are redundantly essential for the TGF-beta-mediated regulation of regulatory T plasticity and Th1 development. *J. Immunol.* 185 842–855. 10.4049/jimmunol.0904100 20548029

[B66] Tang W. B. Ling G. H. Sun L. Liu F. Y. (2010). Smad anchor for receptor activation (SARA) in TGF-beta signaling. *Front. Biosci.* 2 857–860. 10.2741/e147 20515759

[B67] Tang W. B. Ling G. H. Sun L. Zhang K. Zhu X. Zhou X. (2015). Smad anchor for receptor activation regulates high glucose-induced EMT via modulation of Smad2 and Smad3 activities in renal tubular epithelial cells. *Nephron* 130 213–220. 10.1159/000431105 26159183

[B68] Tomoda T. Shirasawa T. Yahagi Y. I. Ishii K. Takagi H. Furiya Y. (1996). Transforming growth factor-beta is a survival factor for neonate cortical neurons: coincident expression of type I receptors in developing cerebral cortices. *Dev. Biol.* 179 79–90. 10.1006/dbio.1996.0242 8873755

[B69] Tsukazaki T. Chiang T. A. Davison A. F. Attisano L. Wrana J. L. (1998). SARA, a FYVE domain protein that recruits Smad2 to the TGFbeta receptor. *Cell* 95 779–791. 10.1016/s0092-8674(00)81701-8 9865696

[B70] Unsicker K. Meier C. Krieglstein K. Sartor B. M. Flanders K. C. (1996). Expression, localization, and function of transforming growth factor-beta s in embryonic chick spinal cord, hindbrain, and dorsal root ganglia. *J. Neurobiol.* 29 262–276. 10.1002/(sici)1097-4695(199602)29:2<262::aid-neu10>3.0.co;2-d 8821181

[B71] van der Wal E. A. Gomez-Pinilla F. Cotman C. W. (1993). Transforming growth factor-beta 1 is in plaques in Alzheimer and Down pathologies. *Neuroreport* 4 69–72. 10.1097/00001756-199301000-00018 8453039

[B72] Vawter M. P. Dillon-Carter O. Tourtellotte W. W. Carvey P. Freed W. J. (1996). TGFbeta1 and TGFbeta2 concentrations are elevated in Parkinsons disease in ventricular cerebrospinal fluid. *Exp. Neurol.* 142 313–322. 10.1006/exnr.1996.0200 8934562

[B73] Villarroel-Campos D. Bronfman F. C. Gonzalez-Billault C. (2016). Rab GTPase signaling in neurite outgrowth and axon specification. *Cytoskeleton* 73 498–507. 10.1002/cm.21303 27124121

[B74] Vogel T. Ahrens S. Buttner N. Krieglstein K. (2010). Transforming growth factor beta promotes neuronal cell fate of mouse cortical and hippocampal progenitors in vitro and in vivo: identification of Nedd9 as an essential signaling component. *Cereb. Cortex* 20 661–671. 10.1093/cercor/bhp134 19587023 PMC2820705

[B75] Wharton K. Derynck R. (2009). TGFbeta family signaling: novel insights in development and disease. *Development* 136 3691–3697. 10.1242/dev.040584 19855012

[B76] Wilson C. Giono L. E. Rozes-Salvador V. Fiszbein A. Kornblihtt A. R. Caceres A. (2020). The histone methyltransferase G9a controls axon growth by targeting the RhoA signaling pathway. *Cell Rep.* 31:107639. 10.1016/j.celrep.2020.107639 32402271

[B77] Wilson C. Nunez M. T. Gonzalez-Billault C. (2015). Contribution of NADPH oxidase to the establishment of hippocampal neuronal polarity in culture. *J. Cell Sci.* 128 2989–2995. 10.1242/jcs.168567 26101350

[B78] Wrana J. L. Attisano L. Wieser R. Ventura F. Massague J. (1994). Mechanism of activation of the TGF-beta receptor. *Nature* 370 341–347. 8047140 10.1038/370341a0

[B79] Wu G. Chen Y. G. Ozdamar B. Gyuricza C. A. Chong P. A. Wrana J. L. (2000). Structural basis of Smad2 recognition by the Smad anchor for receptor activation. *Science* 287 92–97. 10.1126/science.287.5450.92 10615055

[B80] Yi J. J. Barnes A. P. Hand R. Polleux F. Ehlers M. D. (2010). TGF-beta signaling specifies axons during brain development. *Cell* 142 144–157. 10.1016/j.cell.2010.06.010 20603020 PMC2933408

[B81] Zhang X. Huang W. J. Chen W. W. (2016). TGF-beta1 factor in the cerebrovascular diseases of Alzheimers disease. *Eur. Rev. Med. Pharmacol. Sci.* 20 5178–5185. 28051272

