# Supplementary figures and images for: Fine-Tuning the TGFβ Signaling Pathway by SARA During Neuronal Development
Source: Front Cell Dev Biol. 2020 Sep 3;8:550267. doi: 10.3389/fcell.2020.550267 (PMC7494740; doi:10.3389/fcell.2020.550267)

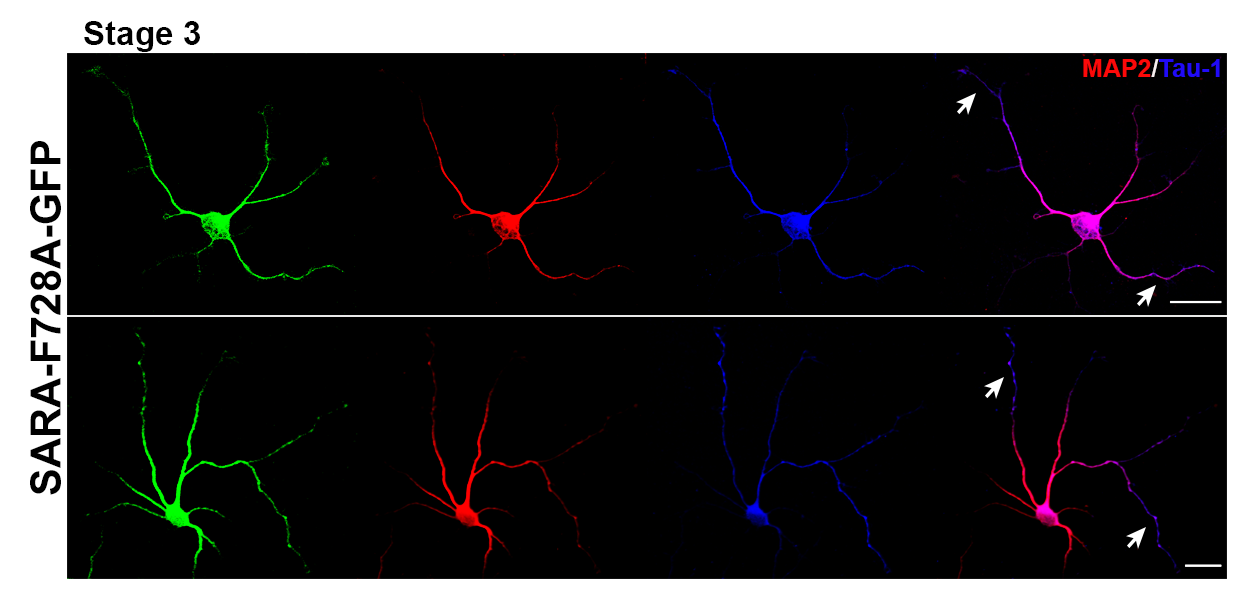

Supplement: FIGURE S1 — Over-activation of the TGFβ pathway by SARA-F728A-GFP generates multi-axonal neurons. (A) Representative images of cultured hippocampal neurons transfected with SARA-F728A-GFP and immunostained to detect MAP2 (red channel) and Tau-1 (blue channel) epitopes. Neurons were transfected after plating and fixed at 3 DIV for IF staining. Arrows show axons in the images. Results represent the mean of three independent cultures (n = 3). Scale bar: 20 μm. [file Image_1.TIF]

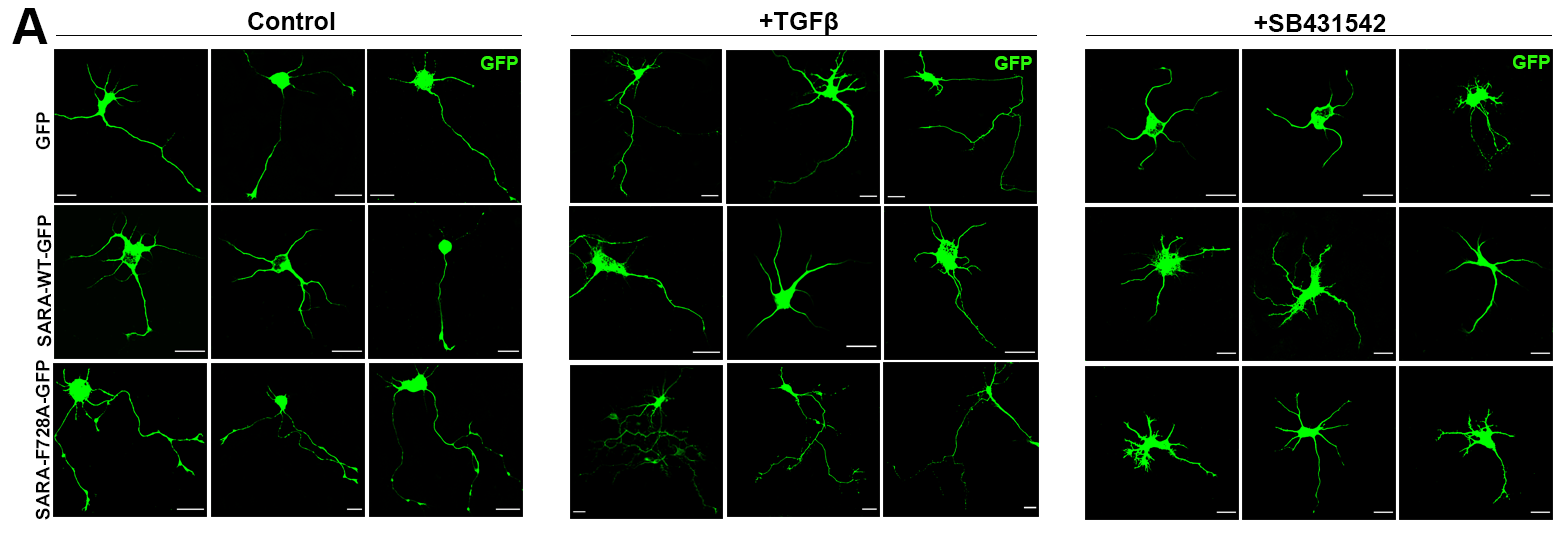

Supplement: FIGURE S2 — Contribution of SARA to TGFβ-dependent axonal growth during neuronal development. (A) Representative images of three DIV hippocampal neurons transfected with GFP, SARA-WT-GFP, or SARA-F728A-GFP and treated with TGFβ (2 ng/ml) or SB431542 (10 μM) (TβRI inhibitor). Results represent different examples from three independent cultures (n = 3). Scale bar: 20 μm. [file Image_2.TIF]
